# Periodontitis, Metabolic Syndrome and Diabetes: Identifying Patients at Risk for Three Common Diseases Using the aMMP-8 Rapid Test at the Dentist’s Office

**DOI:** 10.3390/diagnostics14242878

**Published:** 2024-12-21

**Authors:** Taija Kristiina Hopealaakso, Julie Toby Thomas, Tommi Pätilä, Miika Penttala, Dimitra Sakellari, Andreas Grigoriadis, Shipra Gupta, Timo Sorsa, Ismo T. Räisänen

**Affiliations:** 1 Department of Oral and Maxillofacial Diseases, Head and Neck Center, University of Helsinki and Helsinki University Hospital, 00290 Helsinki, Finland; 2 Department of Pediatric Surgery, New Children’s Hospital, University of Helsinki and Helsinki University Hospital, 00290 Helsinki, Finland; 3 Department of Preventive Dentistry, Periodontology and Implant Biology, Faculty of Health Sciences, Dental School, Aristotle University of Thessaloniki, 541 24 Thessaloniki, Greece; 4 Dental Sector, 424 General Military Training Hospital, 564 29 Thessaloniki, Greece; 5 Oral Health Sciences Centre, Post Graduate Institute of Medical Education & Research (PGIMER), Chandigarh 160012, India; 6 Department of Oral Diseases, Karolinska Institutet, 171 77 Stockholm, Sweden

**Keywords:** periodontitis, active-matrix metalloproteinase-8, aMMP-8, diabetes mellitus, biomarker

## Abstract

**Background/Objectives:** This narrative review paper highlights the multifaceted influence of dysbiotic biofilm, genetic background, host response, and environmental factors on periodontitis. It explores the roles of type I and II diabetes mellitus, gestational diabetes, and metabolic syndrome in the progression of periodontitis, drawing insights from various empirical studies and theoretical perspectives. **Methods**: Relevant articles were sourced using keywords in databases like PubMed/Medline, Science Direct, Scopus, and Google Scholar. Additionally, this review examines the relationship between aMMP-8 levels and increased glycemic states, as well as varying degrees of periodontitis severity. **Results**: The biomarker active-matrix metalloproteinase-8 (aMMP-8), produced by polymorphonuclear leukocytes (PMN), is highlighted as a reliable indicator of ongoing connective tissue degradation. Dysfunctions in PMN activity, accumulation of advanced glycation end products (AGE), and oxidative stress aggravate the periodontal inflammatory response and complications of diabetes. Traditional diagnostics of periodontitis do not provide sufficient information about the current or future disease initiation or activity of periodontitis. **Conclusions**: The implications of this review point to the need for monitoring periodontal health by utilizing innovative strategies like aMMP-8 point-of-care testing, using oral rinse for screening and treatment monitoring, and harnessing the potential of supportive treatments like low-dose doxycycline and light-activated mouth rinses for restoring periodontal health. Its expression in oral fluids is a promising diagnostic tool to differentiate periodontitis from gingivitis and healthy periodontium, especially when associated with systemic diseases, fostering greater collaboration among healthcare professionals.

## 1. Introduction

Diabetes, metabolic syndrome (MetS), and periodontitis are two-way interconnected national common diseases [1,2,3]. Currently, more than 400 million people suffer from diabetes globally, and there are even more who have metabolic syndrome [4,5]. Periodontitis, the multifactorial chronic inflammatory disease that causes progressing destruction of tooth-supporting tissues, is estimated to occur in its severe form in nearly 800 million people [6,7]. Poorly managed diabetes and MetS are known to predispose individuals to periodontitis and contribute to the faster-than-normal progression of periodontitis, making its treatment more difficult [1,2,3]. On the other hand, untreated periodontitis can predispose individuals to diabetes complications, such as retinopathy and nephropathy [1]. Disease-active periodontitis, on the other hand, complicates the diagnosis and treatment of diabetes, but may also contribute to the development of MetS [1,2,3]. Thus, the underdiagnosis and treatment of these diseases can contribute to a significant strain on our health-care resources.

Biomarkers in oral fluids have been diligently studied in the diagnostics of periodontitis [8,9,10]. Active matrix metalloproteinase-8 (aMMP-8) is an important factor in periodontitis and collagenolytic tissue destruction in the periodontium, which total/latent MMP-8 (nowadays abbreviated by MMP-8) lacks [11]. Indeed, aMMP-8 is one of the most researched biomarkers supporting periodontitis diagnostics and treatment monitoring that has shown the most research evidence. In addition to traditional laboratory methods, aMMP-8 analysis is now possible at patient receptions, with an oral fluid point-of-care (POC) test that gives a test result in 5–10 min, the use or interpretation of which does not require the expertise of a dentist, doctor, or healthcare professional. The test is similar in function to a traditional pregnancy or COVID-19 antigen test, and reveals the ongoing active collagenolytic hidden in the patient’s gum tissue, thus indicating periodontitis.

Biomarker testing can contribute to better cooperation between dentistry and general medicine in the diagnosis and treatment of diabetes and periodontitis. At the dentist’s office, in case of high aMMP-8 concentrations (disease activity) in periodontitis patients, the patient’s potential MetS and diabetes risk should be considered and, if necessary, the patient should be referred to a diabetes nurse/doctor for an assessment of the need for treatment. On the other hand, the aMMP-8 test indicates the latent risk of active collagenolytic tissue destruction in the periodontium of a MetS and diabetic patient, and the possible need to refer them to a dentist for further examination. Such oral fluid test diagnostics can have significant positive effects on the better identification of periodontitis and diabetes patients and their treatment.

This narrative review aims to explore relationships between periodontitis, type I and II diabetes mellitus, gestational diabetes, and metabolic syndrome by reviewing various empirical studies and theoretical perspectives. Additionally, this review examines the relationship between aMMP-8 levels and increased glycemic states, as well as varying rates of progression of periodontitis. Relevant articles were sourced using relevant keywords in databases like PubMed/Medline, Science Direct, Scopus, and Google Scholar, without restricting the publication years.

## 2. Periodontitis

The updated clinical classification system of periodontitis from 2018 is based on determining the stage of the disease (severity, extent, and complexity of disease management) and the rate of progression (slow, moderate, rapid) [12,13]. The stage is classified on a scale of I–IV, and the rate of progression on a scale of A–C. The progression rate classification (A–C) is modified by two risk factors, which are smoking and its amount and diabetes, and the associated HbA1c value [12,13]. Sorsa et al. were the first to implement—clinically and successfully—the aMMP-8 biomarker in this new periodontitis stage and progression rate classification [14]. Deng et al. confirmed this functionality of the aMMP-8 biomarker in the new disease classification of periodontitis [15,16].

**Figure 1:**
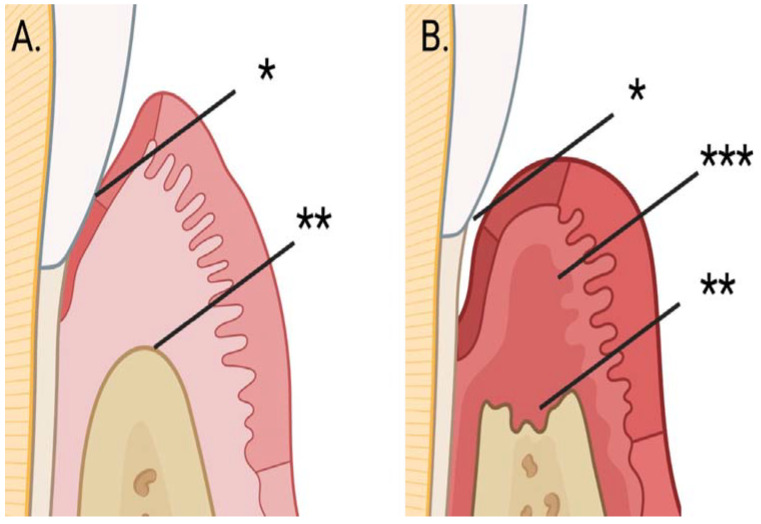
Periodontitis is a condition where the attachment/tooth supporting tissue * around the teeth is damaged, and the jawbone ** that supports the tooth is resorbed. The progression of the disease is associated with active gingivitis ***. The activation of the collagenolytic enzyme MMP-8 to active MMP-8 (aMMP-8) is a key process in periodontitis and an indicator for evaluating its activity. Panels (**A**,**B**) illustrate a healthy periodontium and signs of progression of periodontitis in periodontium. The image was created in BioRender. Pätilä, T. (2024) https://BioRender.com/e55i129. Accessed on 19 December 2024.

Periodontitis (Figure 1) is a multifactorial disease, the occurrence of which is influenced by certain microbes, genetic background, host response, and systemic and local environmental factors [17,18,19,20,21]. Periodontitis is usually preceded by gingivitis [6]. The onset and progression of periodontitis is related to the dysbiosis of the oral microbiome and its interaction with the host’s defense response, which generates inflammation that eventually causes tissue destruction in the periodontium [19,20,21]. The deepened periodontal pockets of individuals with periodontitis mostly contain periodontal pathogens of the so-called red complex, e.g., *Porphyromonas gingivalis*, *Treponema denticola*, and *Tannerella forsythia* [18]. The virulence factors of these anaerobic, gram-negative periodontal pathogens activate the host’s immune response, releasing pro-inflammatory cytokines and chemokines [18]. Matrix metalloproteinases (MMPs) are produced/released in the periodontium by, e.g., macrophages, fibroblasts, junctional epithelial cells, and neutrophils [11]. Production/release is controlled by virulence factors of red complex bacteria and host pro-inflammatory cytokines [11]. MMPs, especially MMP-8, destroy the collagen fibers of the periodontal ligament [11]. The RANK (membrane-bound receptor activator of nuclear factor-κB) ligand on the outer surface of T-helper cells and osteoblasts reacts with the RANK receptor of osteoclast precursor cells, initiating the development and maturation of osteoclasts that destroy alveolar bone [6].

Among the environmental factors, smoking is the most significant cause of periodontitis [22]. Even a small amount of smoking increases the risk of developing periodontitis, and the risk for heavy smokers is up to seven times higher than that of non-smokers [6]. The subgingival biofilm of smokers has more dysbiosis than that of non-smokers [23,24,25,26]. Smoking can affect the host response, especially neutrophils, causing oxidative stress, and making a person more susceptible to periodontitis [27,28]. Smoking also weakens the treatment response of periodontitis [27].

## 3. MetS, Diabetes and Periodontitis

In the activity of the polymorphonuclear leukocyte cells (PMN) of MetS and diabetes patients, dysfunctions possibly related to metabolic changes are found, which affect chemotaxis, phagocytosis and microbicidal properties [29,30]. MetS and diabetes patients with severe periodontitis have impaired chemotaxis of PMN cells and disturbances in PMN cell apoptosis, compared to diabetes patients with mild periodontitis [29,30]. Therefore, PMN cells accumulate in the tissue, causing further tissue destruction when MMP and reactive oxygen molecules are released.

Accumulation of advanced glycation end products (AGEs) in the periodontium of a diabetes patient is also a possible factor contributing to periodontal inflammation [30,31]. When AGE binds to its receptor (RAGE), the production of inflammatory mediators increases, e.g., IL-6, IL-1β and TNF-α [30,31]. The formation of AGEs leads to the production of ROS oxygen radicals (reactive oxygen species), increasing oxidative stress [30,31]. Changes also occur in the endothelial cells that lead to vascular damage, which is related to many complications of diabetes [32]. AGEs also increase the number of PMN cells and worsen bone metabolism so that bone formation and repair is weaker, and less extracellular matrix is formed [30,31].

Moreover, the AGEs-RAGE interaction activates signaling pathways, like nuclear factor-κB (NF-κB) and mitogen-activated protein kinase (MAPK), promoting osteoclast development and increasing receptor activator of nuclear factor kappa-Β ligand (RANKL) expression [33]. NF-κB is a key player in inflammatory diseases, regulating the expression of pro-inflammatory genes, inflammasome regulation, and the survival, activation, and differentiation of innate immune cells and inflammatory T cells [34]. AGEs cause osteoblasts and periodontal ligament cells to express higher RANKL [33,35]. Osteoprotegerin (OPG), a protective factor against bone resorption, acts as a decoy receptor for RANKL [36], where AGEs can decrease OPG expression and affect the RANK-RANKL interaction [37]. Therefore, the AGEs-OPG/RANK/RANKL pathway is crucial in controlling bone loss in chronic inflammatory conditions.

Adipokines, i.e., proteins secreted from adipose tissue, can also predispose individuals to both periodontitis and diabetes [38]. The pro-inflammatory properties of certain adipokines may be particularly important in the exacerbation of periodontal inflammation in overweight or diabetic individuals [30,38]. It should provide a concise and precise description of the experimental results, their interpretation, as well as the experimental conclusions that can be drawn.

MetS and type I and II diabetes patients are characterized by elevated systemic inflammatory markers in the gum tissue and oral fluids, e.g., aMMP-8 [32,39,40,41]. Hyperglycemia, i.e., elevated long-term blood sugar, initiates signaling pathways that increase apoptosis, oxidative stress, and inflammation [29,30,38]. Diabetics and overweight individuals, but also periodontitis patients, have been shown to have elevated serum TNF-α and IL-6 levels, which can induce CRP production, impair intracellular insulin signaling, and lead to insulin resistance for type II diabetes [38]. In patients with periodontitis, systemic inflammation like that predisposes individuals to insulin resistance and further to type II diabetes, and worsens the balance of diabetes treatment [38,39]. On the other hand, periodontal anti-infective treatment reduces serum levels of inflammatory mediators such as IL-6, TNF-α, aMMP-8 in both diabetic and non-diabetic patients [38].

### 3.1. MetS and Type II Diabetes

A person with MetS and/or type II diabetes has three times the risk of developing periodontitis compared to non-diabetics [42,43,44,45]. Glycemic control is a key factor in the increased risk of type II diabetes in periodontitis patients, as periodontitis weakens glycemic control compared to non-periodontitis patients [45]. In periodontitis patients with MetS and type II diabetes, glycemic control progressively worsens, and diabetes-related complications occur more often [45]. On the other hand, according to a study carried out in the United States (NHANES III), adults with an HbA1c level of more than 9% had significantly more severe periodontitis than non-diabetic patients [46].

### 3.2. Type I Diabetes

Type I diabetes increases the risk of developing periodontitis. Ten percent of minors with type I diabetes have been found to have a loss of connective tissue and alveolar bone compared to the control group [47]. According to a more recent study [48], more than 20% of 6 to 8-year-old type I diabetes patients have deepened gum pockets, while the corresponding figure for non-diabetic patients is 8%. Diabetes significantly correlated with periodontal destruction among the younger (<12 years) age group, suggesting it is an important systemic modifier for periodontitis earlier in life [48]. In the older (12–18 years) age group, diabetes was significant for both attachment loss and bleeding, possibly due to the confounding effect of puberty on gingival inflammation [48]. In the combined periodontitis definition of at least two teeth with AL > 2 mm and bleeding at the same sites, the OR for cases over controls was 2.63 [48]. Hence, diabetes significantly impacts periodontal destruction in both 6–11 and 12–18-year-old subgroups [48]. The gingival fluid of type I diabetes patients contains higher PGE2, IL-1b, and aMMP-8 concentrations than in non-diabetic patients, even if the severity of periodontitis in both groups was the same [41,49].

### 3.3. Gestational Diabetes

Gestational diabetes mellitus (GDM) is a growing global health concern, with women with a history of GDM at risk of developing type II diabetes, a risk factor for periodontitis [50]. GDM’s hormonal and metabolic changes cause immune dysregulation, including altered neutrophil function, leading to increased reactive oxygen species and oxidative stress, and causing the destruction of periodontal tissues [51]. GDM, i.e., glucose intolerance first noticed during pregnancy, is predisposed to previous gestational diabetes, the mother’s advanced age, obesity, consanguinity, and polycystic ovary syndrome [50]. Expectant mothers suffering from gestational diabetes have an increased risk of complications during pregnancy, such as pre-eclampsia, and the children who are born have an increased risk of getting sick, e.g., macrosomia, neonatal hypoglycemia, and neonatal heart symptoms [50]. Longer term complications include obesity, impaired glucose tolerance, and diabetes in adulthood or early adulthood [50].

Periodontitis may even more than double the risk of developing GDM [50]. Blood glucose levels and immune responses can alter the oral microbiome, leading to increased glucose in gingival crevicular fluid—a conducive environment for pathogenic bacteria like *Porphyromonas gingivalis*, associated with periodontitis—and accelerating the progression of periodontal disease [38]. MMP-8 and -9 levels in gingival mucosa have been found to be elevated during early pregnancy in women with severe periodontitis, which may be related to the onset of gestational diabetes: in a previous study, it was reported that 14% of pregnancies were diagnosed with GDM, and women with periodontitis stages III and IV had higher concentrations of MMP-8 and -9 in their gingival crevicular fluid (GCF) [52]. The increased level of circulating inflammatory mediators due to gingivitis, combined with gestational diabetes, can act as a starting point for systemic, chronic inflammation [50]. As a result of periodontitis, inflammatory markers, such as interleukins and TNF-α, can be released into the bloodstream, which can cause insulin resistance before long and affect the destruction of beta cells in the pancreas, disrupting glucose metabolism [50]. If left untreated, the condition can lead to gestational diabetes [50].

### 3.4. Treatment of Periodontitis in MetS and Diabetes Patients

The treatment of periodontitis in MetS and diabetes patients requires special attention, as these systemic conditions can affect oral health and slow down the healing of gum disease [1,2,3,38]. Treatment of severe gum disease is started in the same way as for other patients, but MetS and diabetes patients must have closer monitoring and cooperation with healthcare professionals [38,45].

A MetS and diabetes patient’s blood sugar levels must be well controlled, as high blood sugar hinders the healing of periodontitis and can increase the risk of infections [42,45]. Antibiotic therapy may be necessary, and surgical procedures may be more complicated in people with diabetes compared to people with periodontitis without diabetes. Systematic review and meta-analysis conducted by Chen et al. [53] reported that non-infective periodontal therapy significantly improves glycemic control in T2DM patients, particularly those with higher baseline HbA1c levels. This could be due to angiopathy-induced inflammation and impaired healing, allowing periodontal therapy to lower inflammatory markers and potentially improve insulin resistance and glucose metabolism [53].

Managing MetS and diabetes also includes a healthy diet and regular exercise as an essential part of life. These factors also support the success of periodontal treatment. Monitoring periodontal health is important for MetS and diabetics [38,42,43].

It is important for MetS and diabetes patients to take care of good oral hygiene and have regular dental checkups. Supportive treatments can be added to self-care management, such as low-dose doxycycline acting as an aMMP-8 inhibitor in difficult-to-treat periodontitis and light-activated mouth rinses, which, in addition to antibacterial treatment, have an anti-inflammatory effect achieved through mitochondria [54].

## 4. Diagnostics

The traditional diagnostics of periodontitis include measuring the depth of gingival pockets and gingival bleeding, evaluating the clinical attachment tissue level (CAL) and tooth mobility, and examining the bone loss visible on X-rays [12,13]. The traditional diagnostic parameters in use inform doctors about the periodontitis that is already present and the clinical changes caused by it. However, they do not inform doctors with sufficient accuracy about the current or future disease initiation or disease activity of periodontitis [6,49], which has motivated studies on the utilization of biomarkers in the diagnostics and prognostic examination of periodontitis [55].

### 4.1. Biomarkers in Diagnostics

Numerous oral biomarkers have been investigated in relation to periodontitis, and several studies [8,9,10,11] have found elevated levels of aMMP-8, but not total/latent MMP-8, to differentiate periodontitis from both gingivitis and healthy periodontium, and to precede future tissue destruction [14,15,16,56,57,58,59,60,61,62,63,64].

MMPs are enzymes that degrade several extracellular proteins in connection with growth, normal development, and tissue regeneration. According to current knowledge, MMPs together with cytokines cause the destruction of the soft and hard tissue fibers of the periodontium [11]. Eventually, tissue destruction shows clinical signs of periodontitis, such as receding gums, deepened gum pockets, increased tooth mobility and loss [6,12,13]. According to a recent systematic review, aMMP-8 is the most accurate and error-free of the mouthwash biomarkers, but also of the gingival crevicular fluid biomarkers, for periodontitis risk screening [8,9,11].

### 4.2. Different Oral Fluids in Biomarker Diagnostics

Oral fluids (mouthrinse/oral rinse/mouthwash, saliva, gingival crevicular fluid, and peri-implant sulcular fluid) are a rich source of potential biomarkers [8,9,10,11]. They can be collected easily and, if necessary, even without any medical professionals [11]. The collection can be performed non-invasively, i.e., without a risk of causing a bacteremia to a patient that is an advantage among patients with immunosuppression [11]. Collection of oral fluids is painless and rarely causes any discomfort to patients in contrast to, for example, collection of blood samples. Mouthrinse and saliva are whole-mouth oral fluids that provide information at the patient level, while gingival crevicular fluid and peri-implant sulcular fluid are used for site-specific biomarker diagnostics [11].

GCF can be considered as a presentation of the current inflammatory situation of periodontal pocket. As such, GCF provides information about the local periodontium of the tooth, and its volume and flow increase significantly in disease compared with a healthy sulcus [65]. GCF can be easily collected for biomarker analysis with absorbent filter paper strips, capillary tubing, or micropipettes from a site or many sites of the tooth [66]. Peri-implant sulcular fluid is comparable to gingival crevicular fluid in its composition, qualities, and features [11].

Mouthrinse is a direct derivative of GCF from the periodontal pockets of all teeth and thus, presents information of the current situation of the periodontium in the whole mouth [11,67]. Thus, mouthrinse is the preferable collection method for whole-mouth biomarker analysis instead of collecting gingival crevicular fluid site by site of all teeth. The mouthrinse collection is based on pre-rinsing to remove saliva and other interfering subjects from the mouth, which is followed by a small wait, after which the actual collection is performed by rinsing a test fluid, usually with standardized volume and rinsing time [11]. That ensures some degree of standardization to the mouthrinse (i.e., whole mouth gingival crevicular fluid) collection that, for example, collecting saliva is lacking. Some patients may suffer from xerostomia or hyposalivation, which effects saliva secretion volumes, and mouthrinse collection is much easier than saliva collection for these patients [68].

The mouthrinse collection method also minimizes the contamination of mouthrinse by saliva. Saliva is a unique oral fluid that consists mostly of the secretions of major salivary glands and in small amounts from the minor salivary glands [66]. Saliva also contains components from other sources that are non-salivary glands, such as gingival crevicular fluid, epithelial cells shed from mucosal surfaces, bacteria, viruses, food debris, etc. [66]. Thus, there are multiple potential confounding factors that may have an effect on the composition of saliva, if one is interested in biomarkers originating from gingival crevicular fluid. That underscores the importance of utilizing mouthrinse instead of saliva in such cases.

### 4.3. aMMP-8 Diagnostics

Matrix metalloproteinase-8 (MMP-8), produced by neutrophils, in its activated form, i.e., aMMP-8, is involved in the breakdown of type I collagen in connective tissues [11]. Its activity is linked to periodontal disease progression, and its detection is valuable in diagnosing periodontal and inflammatory diseases, due to its specificity and role in tissue degradation, which total/latent MMP-8 (nowadays abbreviated by MMP-8) lacks [56,57,58]. aMMP-8 levels in body fluids have been found to differentiate active and stabilize periodontal sites, with elevated levels indicating inflammation and collagen breakdown, and low levels indicating stability, thus, guiding targeted therapy [11]. Diagnostic effectiveness of aMMP-8 in different oral fluids have been estimated in previous meta-analysis to range between 63.0–76.7% (median sensitivity) and 70.5–92.0% (median specificity) [8,9,69]. Furthermore, mouthrinse as the derivative of GCF has been found to be a more accurate oral fluid in aMMP-8 diagnostics than saliva [14,16]. Previous studies have also found that aMMP-8 levels may be able to distinguish the stages of periodontitis (severity of periodontitis) [14]. High aMMP-8 levels are linked to systemic inflammatory conditions like MetS and diabetes, with periodontitis as a potential risk factor [40,70]. Monitoring these levels can help identify inflammatory burdens and periodontal care needs [55]. aMMP-8 helps differentiate between peri-implant mucositis and peri-implantitis, with higher levels in peri-implantitis, due to increased collagen breakdown [11]. This helps clinicians adjust treatment plans and ensure the health of peri-implant tissues, especially in patients with systemic diseases [55].

In addition to laboratory methods, there is a non-invasive (non-bacteremia) chair-side immunotest-stick for measuring aMMP-8 concentration, which functions similarly to pregnancy and COVID-19 antigen-type lateral-flow tests (point-of-care test, POCT). It is based on measuring the concentration of tissue-destroying catalytically components and collagenolytic aMMP-8 biomarkers from oral fluids (mouthrinse/oral rinse, gingival crevicular fluid, and peri-implant sulcular fluid) [14,15,16]. Combined with a computer reader (PerioSafe/ORAlyzer combination for mouthrinse/oral rinse in whole-mouth diagnostics, or ImplantSafe/ORAlyzer for gingival crevicular fluid/peri-implant sulcular fluid in site-specific diagnostics) [11], the test screens quantitatively reveals the starting, developing, and ongoing collagenolytic periodontal attachment tissue destruction and loss of teeth, i.e., periodontitis, by measuring aMMP-8 levels in oral fluids in 5–10 min [11,14,15,16]. A recent systematic review did not find a significant difference between cut-offs of aMMP-8 concentrations of 10, 20 and 25 ng/mL in mouthrinse/oral rinse to distinguish periodontitis from health [69]. However, two recent studies suggest 20 ng/mL to be the most optimal and accurate, decreasing the risk of false positives and false negatives (also the cut-off of PerioSafe and ImplantSafe aMMP-8 tests) [70,71].

**Figure 2:**
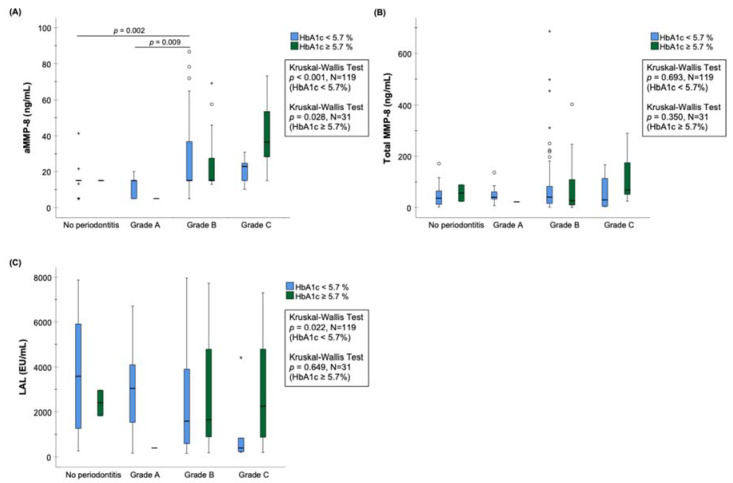
Box-plot plots of (**A**) active MMP-8 (aMMP-8) (ng/mL) lateral-flow oral rinse point-of-care measurement technology (POCT) (PerioSafe/ORAlyzer combination); (**B**) total (latent and active) MMP-8 saliva test (ng/mL) (ELISA, Quantikine, R&D Systems, Minneapolis, MN, USA); and (**C**) lipopolysaccharide (LPS) activity measurement with LAL bacterial endotoxin assay (EU/mL), grouped according to the risk category of periodontitis progression among 150 Greek adults. Marked in the figure, statistically significant differences in aMMP-8 and total MMP-8 concentrations and LAL analysis [73] were calculated using the Kruskal-Wallis test and pairwise post hoc test (Dunn–Bonferroni test). Asterisk (*) and circle (o) represent outliers of more than 3 times the interquartile range and between 1.5 and 3 times the interquartile range, respectively. Adapted from article [64] by Gupta et al. (panel **A**) under the terms of the Creative Commons Attribution (CC BY) license https://creativecommons.org/licenses/by/4.0/. Accessed on 19 December 2024.

Studies have shown that aMMP-8 concentration is also elevated in oral fluids in type I and II and MetS diabetes patients, as well as in women with gestational diabetes [31,40,41,72]. In addition to periodontitis, the concentration of aMMP-8 in mouthwash is also associated with an increased HbA1c value [11,40,41]. Figure 2 shows the relationship between the risk class of periodontitis progression in 150 Greek adults [14,64] and oral rinse aMMP-8, salivary total MMP-8, and salivary LPS/(LAL) activity [73] measurements, divided according to patient HbA1c. There was a significant association between periodontitis risk categories and aMMP-8, where the highest aMMP-8 concentrations were in those patients with elevated HbA1c values. Total MMP-8 measurement showed no significant association with periodontitis or prediabetes. LPS activity measurement had a significant association with periodontitis only in the HbA1c < 5.7% group (downward trend). The aMMP-8 measurement proved to be a clearly more accurate biomarker compared to total MMP-8 and LPS activity measurements.

The result in Figure 2 expands previous studies on the use of the aMMP-8 oral rinse test in identifying the potential risk of (pre)diabetes in periodontitis patients at the dentist’s office [11]. The idea is that, due to the two-way connection between periodontitis and diabetes, a dentist or dental hygienist could refer their own periodontitis patients, who they suspect have an increased risk of diabetes, to a diabetes nurse or doctor for a more accurate blood sugar test and diagnosis and to assess the need for further treatment (this applies to MetS, as well) [2,3]. The results also seem positive regarding diagnostics that take place in the other direction, i.e., the use of aMMP-8 measurements by doctors and other healthcare professionals in identifying periodontitis, and referring patients to a dentist for a more detailed periodontal examination. Future studies will provide more information about the possible benefits of aMMP-8 point-of-care testing in this regard.

## 5. Conclusions

This comprehensive review explored the relationship between periodontitis, type I and II diabetes mellitus, gestational diabetes, and metabolic syndrome, which have a rich molecular interrelationship. Periodontitis is additionally associated in a similar way with several other systemic diseases, such as cardiovascular diseases, respiratory and kidney diseases, Alzheimer’s disease, and cancers. As a result of a bacterial infection in the gum pocket, proinflammatory cytokines and aMMP-8 are produced and released, which, when they end up in the bloodstream, cause inflammation elsewhere in the body [6,11]. The resulting inflammation, proteolysis, and low-grade inflammation, weaken the treatment balance of systemic diseases and the immune response [29,31,43,44,74,75,76]. Especially in patients suffering from systemic diseases, but also in other patients, the progressive gum disease periodontitis should be diagnosed and treated as early as possible, in the most effective way possible [77,78,79,80,81,82].

In previous studies, aMMP-8 analysis has found a significant connection with periodontitis and its severity and risk of its progression categories [11,14,15,16]. Previous studies have also reported elevated aMMP-8 levels among type I and II, gestational, and MetS diabetes patients [11,31,40,41,72]. However, as this review (see Figure 2) shows, there was not found an association between lipopolysaccharide (LPS) activity measurement by LAL bacterial endotoxin assay and grade of periodontitis (i.e., rate of its progression) that could be utilized as accurately as aMMP-8 in periodontitis diagnostics in patients with normo- and hyperglycemia. This suggests that although oral hygiene and bacterial biofilm play a role in periodontitis etiology and oral health, the progression of periodontitis is not associated with LPS activity but elevated aMMP-8 levels. Nevertheless, more studies are required to confirm this in other populations because of the cross-sectional analysis in this study.

Oral fluid analysis based on the aMMP-8 biomarker offers a promising new option for early identification of previously undiagnosed periodontitis and its risk patients in various treatment points, such as the doctor’s office [11,14,15,16,72]. On the other hand, high concentrations of aMMP-8 in oral fluids in patients with periodontitis may indicate an increased risk of (pre)diabetes, and the need for dentists to refer such patients to a diabetes nurse or doctor’s office for more detailed follow-up examinations. Furthermore, aMMP-8 analysis can be used in monitoring the treatment effect of periodontitis among both systematically healthy as well as patients with diabetes and MetS [71,72,83]. New studies should be developed and aimed at exploring these new alternatives for utilizing aMMP-8 diagnostics at the dentist’s and at the doctor’s office in different and large enough cohorts.

## Data Availability

The data supporting the findings of this study are available upon reasonable request from the corresponding author. The data are not publicly available due to privacy and ethical restrictions.
